# Mendelian randomization supports genetic liability to hospitalization for COVID-19 as a risk factor of pre-eclampsia

**DOI:** 10.3389/fcvm.2024.1327497

**Published:** 2024-03-08

**Authors:** Weizhen Wu, Junning Zhang, Yizhuo Qiao, Yuehan Ren, Xuezhi Rao, Zhijie Xu, Baoxing Liu

**Affiliations:** ^1^ Graduate School, Beijing University of Chinese Medicine, Beijing, China; ^2^ Department of Andrology, China-Japan Friendship Hospital, Beijing, China; ^3^ Department of Gynecology, Xiyuan Hospital of China Academy of Chinese Medical Science, Beijing, China

**Keywords:** Mendelian randomization, COVID-19, pre-eclampsia, eclampsia, EBI, GWAS

## Abstract

**Background:**

Pre-eclampsia and eclampsia are among the major threats to pregnant women and fetuses, but they can be mitigated by prevention and early screening. Existing observational research presents conflicting evidence regarding the causal effects of coronavirus disease 2019 (COVID-19) on pre-eclampsia risk. Through Mendelian randomization (MR), this study aims to investigate the causal effect of three COVID-19 severity phenotypes on the risk of pre-eclampsia and eclampsia to provide more rigorous evidence.

**Methods:**

Two-sample MR was utilized to examine causal effects. Summary-level data from genome-wide association studies (GWAS) of individuals of European ancestry were acquired from the GWAS catalog and FinnGen databases. Single-nucleotide polymorphisms associated with COVID-19 traits at *p* < 5 × ^−8^ were obtained and pruned for linkage disequilibrium to generate instrumental variables for COVID-19. Inverse variance weighted estimates were used as the primary MR results, with weighted median and MR-Egger as auxiliary analyses. The robustness of the MR findings was also evaluated through sensitivity analyses. Bonferroni correction was applied to primary results, with a *p* < 0.0083 considered significant evidence and a *p* within 0.083–0.05 considered suggestive evidence.

**Results:**

Critical ill COVID-19 [defined as hospitalization for COVID-19 with either a death outcome or respiratory support, OR (95% CI): 1.17 (1.03–1.33), *p* = 0.020] and hospitalized COVID-19 [defined as hospitalization for COVID-19, OR (95% CI): 1.10 (1.01–1.19), *p* = 0.026] demonstrated suggestive causal effects on pre-eclampsia, while general severe acute respiratory syndrome coronavirus 2 infection did not exhibit a significant causal effect on pre-eclampsia. None of the three COVID-19 severity phenotypes exhibited a significant causal effect on eclampsia.

**Conclusions:**

Our investigation demonstrates a suggestive causal effect of genetic susceptibility to critical ill COVID-19 and hospitalized COVID-19 on pre-eclampsia. The COVID-19 severity exhibited a suggestive positive dose–response relationship with the risk of pre-eclampsia. Augmented attention should be paid to pregnant women hospitalized for COVID-19, especially those needing respiratory support.

## Introduction

From December 2019, the novel coronavirus, also known as severe acute respiratory syndrome coronavirus 2 (SARS-CoV-2), triggered a worldwide pandemic, leading to severe pneumonia in some populations. As of August 2023, the most recent statistics from the World Health Organization indicate that the coronavirus disease 2019 (COVID-19) brought on by this new coronavirus has caused 760 million infections and 6.9 million fatalities, posing a major threat to global health (available online: https://covid19.who.int/). Understanding the relationships between COVID-19 and other diseases helps inform disease prevention strategies. Due to the physiological upregulation of angiotensin-converting enzyme 2 (ACE2) receptors, pregnant women are more susceptible to COVID-19 infection (1). In addition, as pregnancy progresses, hormone levels in pregnant women, including steroids, estrogen, and progesterone, increase beyond normal levels to shift the immune system bias from inflammatory responses to anti-inflammatory responses to protect the fetus. The elevation in progesterone levels can induce lymphocyte synthesis of the progesterone-induced binding factor (PBIF), which elevates during pregnancy and significantly decreases after birth (2, 3). The increased PBIF promotes CD4+ T-cell differentiation into T-helper cells by inducing the secretion of anti-inflammatory cytokines such as IL-4, IL-5, and IL-10. To prevent rejection of the fetus, adaptive immunity (including humoral immunity and cellular immunity) is inhibited during pregnancy (4). These above changes in hormones and immunity render pregnant women more susceptible to COVID-19 infection during pregnancy. Furthermore, based on observational studies, COVID-19 infection has been related to increased risks of various pregnancy-related conditions including hypertensive disorders of pregnancy [including eclampsia and pre-eclampsia (PE)], preterm birth, severe neonatal morbidity, and stillbirth (5–7).

PE is characterized by hypertension developing after 20 weeks of gestation, along with proteinuria, neurological complications, coagulation abnormalities, and uteroplacental disorders, including placental abruption and fetal growth restriction (8). Eclampsia is characterized by seizures or coma in women with PE that cannot be attributed to other causes. These are the two most severe complications of pregnancy and are the leading causes of maternal mortality during pregnancy and childbirth (9–11). PE affects 5%–7% of pregnancies (12) and results in an estimated 62,000–77,000 deaths annually (11). In terms of pathophysiology, pre-eclampsia is caused by placental dysfunction due to various factors, leading to syncytiotrophoblast stress (13, 14). The stressed syncytiotrophoblasts release pro-inflammatory factors, reactive oxygen species, and anti-angiogenic substances into the maternal circulation (13, 15–17), resulting in maternal vascular endothelial dysfunction and multi-organ dysfunction, including decreased vasodilation, thrombosis, systemic inflammation, and other complications. This further leads to hypertension, thrombocytopenia, liver and kidney damage, among others (18–20). Pregnant women with PE often die from complications including hemolysis, elevated liver enzymes, low platelet count (HELLP) syndrome, coma, central nervous system hemorrhage, disseminated intravascular coagulation, cardiac arrest, and pulmonary edema. PE is also an independent risk factor for stroke during pregnancy (21). The fetus is affected by complications like placental abruption and placental insufficiency, with a 3.12-fold increased risk of death compared to non-pre-eclamptic pregnancies. Neonatal mortality is also 2.7 times higher than term pregnancies due to high preterm birth rates (11).

Most observational studies conclude that COVID-19 increases the risk of PE (22–24). A meta-analysis revealed that women infected with SARS-CoV-2 during pregnancy had an increased risk of developing PE compared to uninfected pregnant women (OR, 1.62; 95% CI, 1.45–1.82) (25). However, another meta-analysis found the association to be insignificant, with the incidence of PE lower in infected European pregnant women compared to other ethnicities (26). A few observational studies published later also supported the insignificance (27–29). In most of these observational studies, COVID-19 severity was not stratified, and its proportion was not reported. The study populations may have differed in distributions of COVID-19 severity, which could potentially explain the inconsistent findings. Stratifying COVID-19 severity and adopting research methodology with less bias to investigate the relationship between COVID-19 and pre-eclampsia has become an imperative issue to be addressed.

A method employed in epidemiology called Mendelian randomization (MR) uses genetic variations as instrumental variables (IVs) to examine possible causal links between exposures and outcomes (30). MR mimics a randomized controlled trial, whereby alleles are randomly allocated based on Mendel's second law of segregation, realizing causal inference free from biases like confounding and reverse causation that affect traditional observational studies (31). During meiosis, alleles segregate and assort independently of environmental and disease factors, establishing a random distribution of genetic variation at conception, which is unrelated to subsequent exposures or disease processes (32). Thus, MR studies using genetic variants as proxies for exposures offer stronger evidence for causality compared to retrospective analyses prone to confounding. Overall, MR maximizes control over confounding and reverse causation inherent in conventional epidemiological studies by exploiting the random assortment of genotypes to strengthen causal inferences between exposures and outcomes (33). Since Mendelian randomization has less bias, it is often used to validate findings from observational studies. For example, the study “COVID-19 is associated with the risk of cardiovascular disease death: A two-sample Mendelian randomization study” used MR to validate that the susceptibility to COVID-19 infection and cardiovascular disease mortality has a causal relationship at the genetic level (34).

Mendelian randomization studies have revealed causal relationships between COVID-19 and hypertensive disorders of pregnancy (35). PE belongs to the category of hypertensive disorders of pregnancy, and causal relationships may also exist at the genetic level between COVID-19 and PE. Given the strengths of MR and the research foundation on COVID-19 and hypertensive disorders of pregnancy, we performed two-sample MR analyses using genome-wide association study (GWAS) summary data on three COVID-19 infection severity phenotypes and PE and eclampsia. The goal was to uncover potential causal relationships between COVID-19 and PE and eclampsia and provide novel evidence for this research area.

## Materials and methods

### Study design

Our study is based on summary data from published GWAS studies. To reduce endogeneity bias caused by sample overlap, we need to use samples from two independent GWAS studies, so a two-sample MR approach is utilized. In contrast, the endogeneity bias caused by sample overlap in one-sample MR cannot be overcome by using summary data. Instead, it requires individual-level data from the original GWAS.

This study used two-sample MR to assess the causal effect of three types of COVID-19 severity traits on PE and eclampsia. MR uses genetic variants associated with the exposure and outcome as instrumental variables (often single-nucleotide polymorphisms, SNPs) to determine the causal relationship to test conclusions drawn from previous observational studies.

Qualified IVs in MR need to meet three basic assumptions (36): (1) the IVs are strongly related to exposure; (2) the IVs are independent of potential confounders; and (3) the IVs are only indirectly related to the outcome through the exposure.

The requirement for SNPs to be strongly associated with the exposure in selecting instrumental variables is because the *α*-value needs to be corrected for multiple comparisons in GWAS studies examining the association between a phenotype and multiple SNPs, resulting in a smaller adjusted *p*-value. Typically, 5 × 10^−8^ is used as the genome-wide significance threshold, so the corresponding *p*-value needs to be very small to establish the SNP–exposure association. Only SNPs meeting this criterion can represent susceptibility to the exposure. If the instrumental variables are associated with known confounders, they may indirectly affect the outcome through those confounders. This makes it impossible to confirm whether the exposure is an independent factor influencing the outcome. If the instrumental variables are directly associated with the outcome, the causal direction cannot be determined.

**Figure 1:**
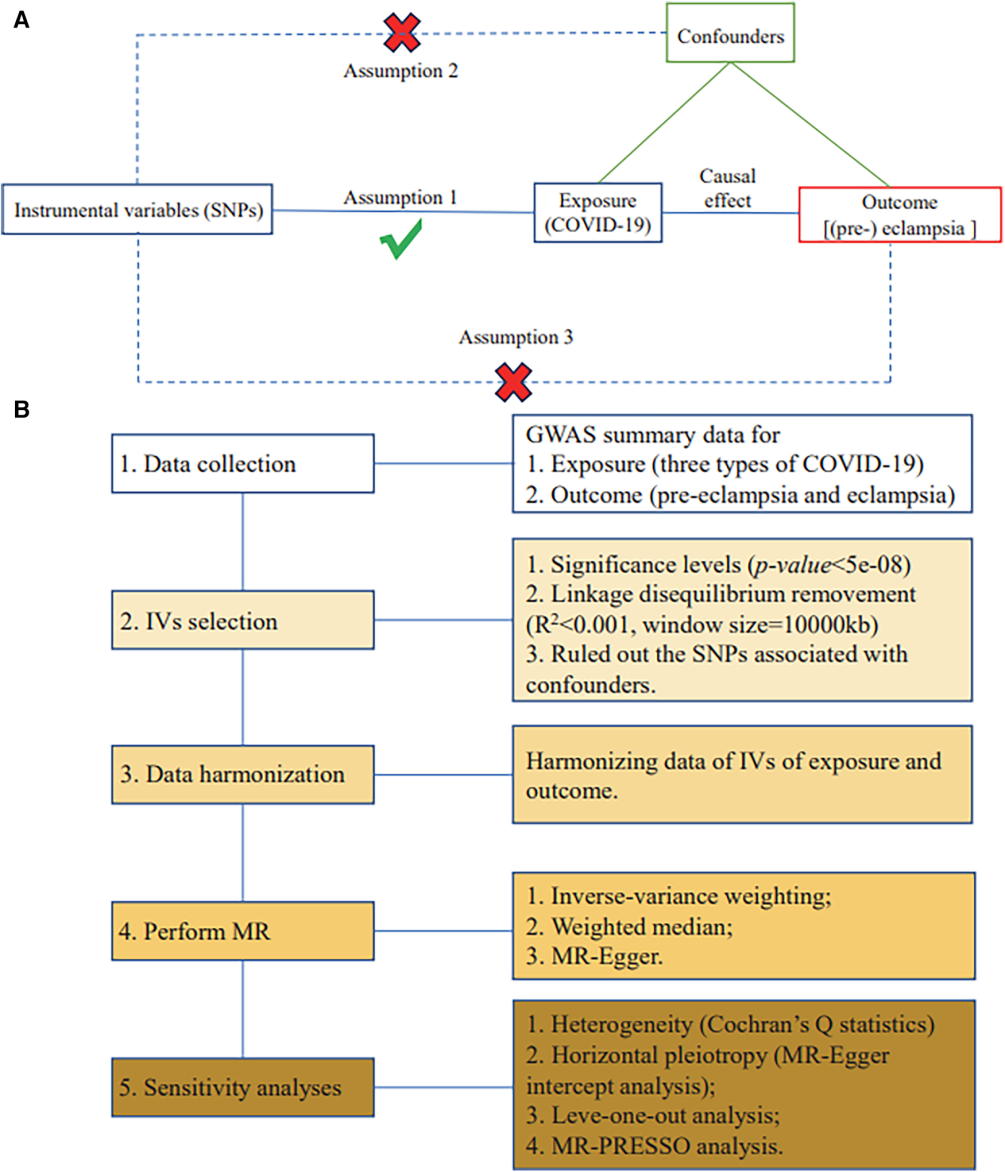
MR assumptions and study design. (**A**) Three fundamental assumptions of MR. (**B**) Flowchart of the current two-sample MR study.

MR assumptions are illustrated in Figure 1A. The flowchart is exhibited in Figure 1B.

### Data sources

**Figure 2:**
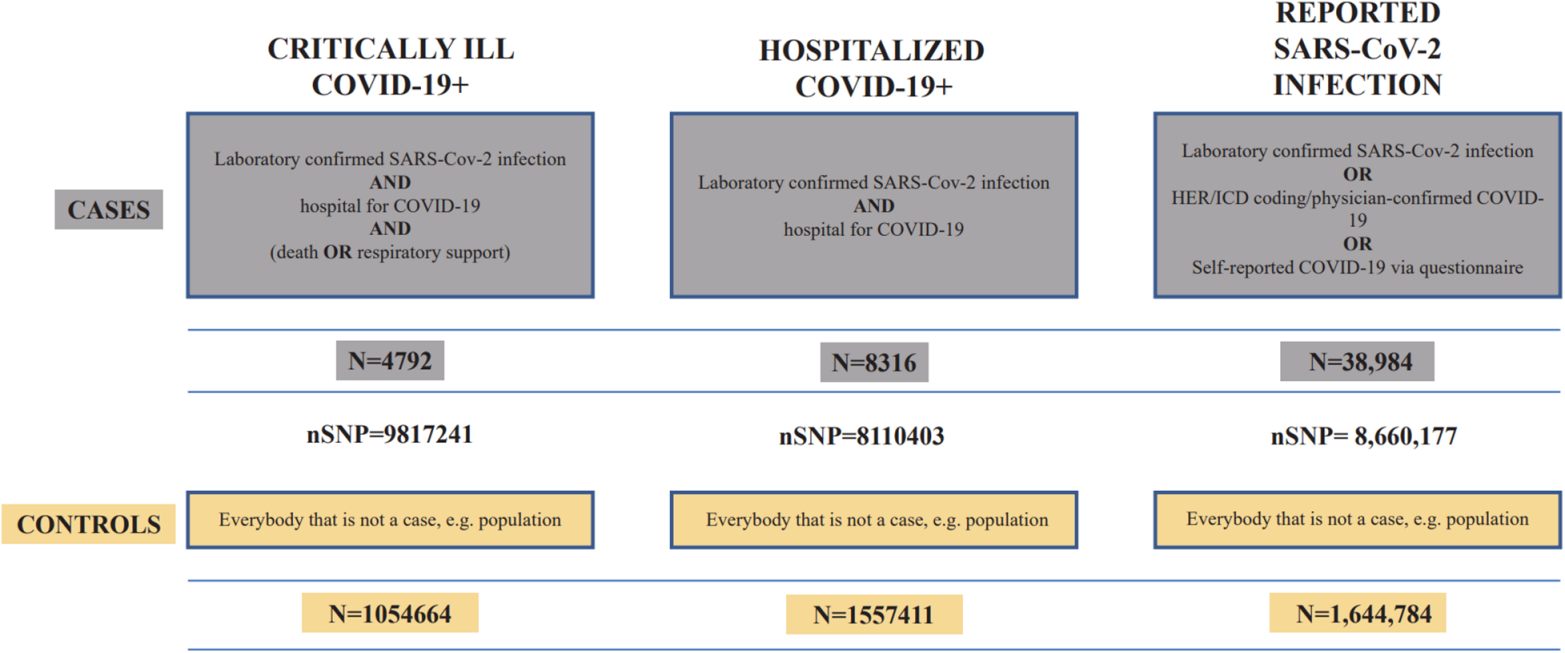
Details of the three COVID-19 GWAS summary datasets.

The exposure data were based on studies published by the COVID-19 Host Genetics Initiative (37), containing three phenotypes of coronavirus infection with varying severity, all derived from individuals of European ancestry. The dataset IDs for critical ill COVID-19, hospitalized COVID-19, and general SARS-CoV-2 infection were “GCST011078,” “GCST011083,” and “GCST011073,” respectively. The data were downloaded through the GWAS catalog (https://www.ebi.ac.uk/gwas/). Figure 2 demonstrates the details of COVID-19 data.

We downloaded summary data on traits characterized as “pre-eclampsia” and “eclampsia” from the GWAS in Europe from the FinnGenn database (38) (https://www.finngen.fi/en/access_results). The dataset IDs for pre-eclampsia and eclampsia were “finngenn_R5_O15_PRE-ECLAMPSIA” and “finngenn_R5_O15_ECLAMPSIA,” respectively, all derived from the female population of European ancestry. PE was confirmed through ICD codes of hospital discharge or cause of death (ICD-10 code O14, ICD-9 codes 642[4-5], and ICD-8 code 6370[349]), and eclampsia was confirmed through ICD-10 code O15 and ICD-9 code 6426. For PE, there were 3,556 cases, 114,735 controls, and 16,379,670 SNPs. For eclampsia, there were 290 cases, 114,735 controls, and 16,379,586 SNPs.

Both the exposure and outcome data were filtered based on genotype quality control before being released.

### Selection and evaluation of SNPs

A *p*-value threshold of <5 × 10^−8^ was utilized as the criterion for selecting SNPs with a strong correlation with COVID-19.

To ensure the independence of the IVs, we performed clumping to exclude linkage disequilibrium (LD) with *r*^2^ < 0.001 within a physical distance of 10,000 kb. We further harmonized the effect alleles in the exposure and outcome datasets and confirmed the absence of palindromic SNPs. The selected SNPs were searched in the PhenoScanner database (http://www.phenoscanner.medschl.cam.ac.uk/) for associations with other potential confounders such as obesity, smoking, diabetes (type 1 and type 2), hypertension, chronic kidney disease, systematic lupus erythematosus, and anti-phospholipid syndrome (39).

### MR analysis

Before the MR analysis, we harmonized the data of the exposure and outcome datasets (see Supplementary Table S1 for the harmonized dataset). For the estimation of causal effects, the random-effects inverse variance weighting (IVW) method was used as the primary MR approach, while the weighted median and the Mendelian randomization-Egger (MR-Egger) approaches were used as auxiliary methods:

1. IVW assesses causality by merging the ratio estimates of selected SNPs (40).
2. Weighted median produces unbiased estimates, even if up to 50% of IVs are invalid (41).
3. MR-Egger determines causality through the slope coefficients of Egger regressions and can also detect small study biases (42).

IVW is the most commonly used method. It requires SNPs to fully satisfy the three assumptions for valid instrumental variables to obtain unbiased causal estimates. Among the three methods, IVW has the greatest statistical power when the IVs do not exhibit horizontal pleiotropy or heterogeneity. MR-Egger is used as the primary analysis when the IVs demonstrate horizontal pleiotropy (43). The weighted median method estimates causality based on the majority of genetic variants and is used as the primary analysis when the IVs exhibit heterogeneity.

Moreover, to evaluate the correlation of these IVs with exposure, we assessed them by *R*^2^ values and *F*-statistics. The *F*-statistic represents the statistical power of the association between an SNP and exposure. A value below 10 indicates a weak instrument that is inadequate to represent the exposure and should be removed from the instrumental variable set. The *F*-statistic serves as another test of the ability of candidate instrumental variables to capture the exposure after the *p* < 5 × 10^−8^ filter (43). The formulas are as follows:

$$R^{2}=2\times (1-\mathrm{MAF})\times (\mathrm{MAF})\times {\left(\frac{\beta }{SE\times \sqrt{N}}\right)}^{2}$$

$$F=\frac{N-k-1}{k}\times \frac{R^{2}}{1-R^{2}}$$

where MAF denotes the minor allele frequency, *β* is the effect size, *R*^2^ is the extent of IV explaining the exposure, SE denotes the standard error, *N* is the sample size, and *K* denotes the number of SNPs.

### Sensitivity analysis

To evaluate the robustness of statistically significant causal relationships, several sensitivity analyses were employed. We assessed heterogeneity through Cochran's *Q*-test (44). MR-Egger's intercept analysis was utilized to estimate horizontal pleiotropy, which implies that certain IVs affect the outcome through associations with confounders other than the exposure. Simply screening for associations between instrumental variables and known confounders may miss some confounders, while a non-significant finding from MR-Egger suggests that there is no meaningful horizontal pleiotropy (42). A leave-one-out analysis was performed, where SNPs are eliminated one at a time to examine whether a single SNP can determine or reverse the results alone. MR Pleiotropy Residual Sum and Outlier (MR-PRESSO) was used to detect the effects of outliers (45). The causal effect of each SNP on PE and eclampsia was calculated by the Wald ratio method, and a threshold was defined as *p* < 0.05/*n* (*n* refers to the number of SNPs) according to Bonferroni correction, where 0.05/*n* < *p* < 0.05 was considered suggestive of an effect. We also applied the Steiger test to examine whether the IVs demonstrate stronger correlations with the exposure than with the outcome. A *p*-value <0.05 indicates the absence of reverse causation in the current MR analyses (46). Reverse causality, referred to here, means that the overall association between the instrumental variables and the outcome is stronger than with the exposure. This violates the exclusion restriction principle for valid instrumental variables, making it impossible to determine the causal direction.

### Statistical analysis

We used the “TwoSampleMR” R package (v0.5.7) in R software (v4.3.1) (http://www.r-project.org) to perform MR analyses and sensitivity analyses. The Steiger test was conducted through the “r.test” function available in the “psych” R package (v2.3.6). For MR analyses, the *p*-value of IVW and the directional consistency across all three MR methods are crucial for establishing causality. To account for multiple testing, the Bonferroni-corrected threshold of *p*-value <0.0083 [0.05/6 (three exposures and two outcomes)] was applied as a preset threshold. A *p*-value <0.0083 is indicative of significance, and a *p*-value between 0.0083 and 0.05 is considered suggestive evidence. Due to the limited sample size of the pre-eclampsia and eclampsia case groups, the number of IVs may result in *p*-values not reaching the corrected *α*-value; however, *p*-values between 0.05 and the corrected *α*-value hold potential statistical significance because if this study only examined the relationship between one phenotype pair, *p* < 0.05 would be statistically significant. Therefore, we refer to previous studies to define such *p*-values as a “suggestive causal effect” (47–49). For analyses regarding heterogeneity and horizontal pleiotropy, a *p*-value <0.05 is considered significant.

## Results

### Genetic IVs for COVID-19

SNPs strongly associated with each COVID-19 severity trait (*p*-value for association hypothesis <5 × 10^−8^) were first selected from the COVID-19 GWAS data. After clumping, only one representative SNP was retained from each linkage disequilibrium region. This resulted in eight, seven, and seven SNPs strongly associated with critical ill COVID-19, hospitalized COVID-19, and SARS-CoV-2 infection, respectively. After removing SNPs not overlapping with the pre-eclampsia GWAS data, seven, six, and seven SNPs were retained, respectively. Harmonization of the effect alleles of selected SNPs overlapping between the two GWAS samples revealed consistency, and no palindromic SNPs were identified. No SNP was found to be strongly associated with known confounders. Therefore, the final instrumental variables consisted of seven, six, and seven SNPs for critical ill COVID-19, hospitalized COVID-19, and SARS-CoV-2 infection, respectively. This process satisfies the three assumptions of Mendelian randomization instrumental variable selection, as explained in the response to Comment 7 (strong relation to exposure, independence from confounders, indirect relation to outcome). Information on allele frequencies, effect estimates, standard errors, and *p*-values for these SNPs on exposure (COVID-19) and outcome (pre-eclampsia and eclampsia) is provided in Supplementary Table S1. Furthermore, we calculated *R*^2^ for the final IVs included in the analysis (the degree to which the IVs explain the exposure) to derive *F*-statistics, all of which were greater than 10 (Supplementary Table S1). No SNPs were excluded for association with confounders.

### Causal effects of critically ill COVID-19 on pre-eclampsia and eclampsia

**Figure 3:**
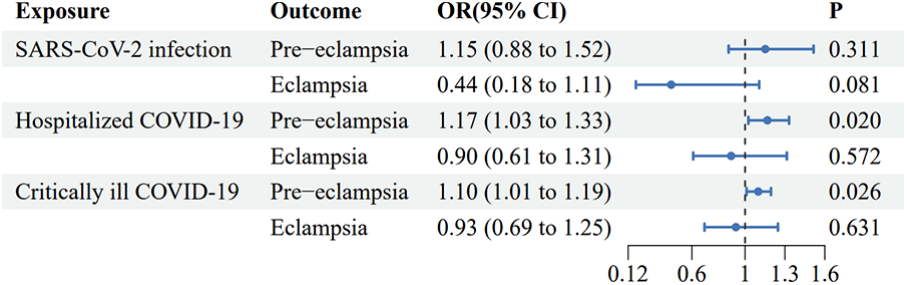
Forest plot of inverse variance weighted Mendelian randomization analyses of three types of COVID-19 traits on the risk of pre-eclampsia and eclampsia. CI, confidence interval; OR, odds ratio.

Using IVW analysis as the primary approach, we found evidence of a suggestive causal effect of genetic liability to critical ill COVID-19 infection on the risk of PE [OR (95% CI): 1.17 (1.03–1.33), *p* = 0.020], with odds ratios greater than 1 across the other MR methods. An OR of 1.17 indicates that the risk of pre-eclampsia is 1.17 times higher in the critically ill COVID-19 group compared to the uninfected group. The 95% CI of 1.03–1.33 means there is a 95% probability that the true OR lies between 1.03 and 1.33 (similarly hereafter). However, IVW analysis indicated no significant causal effect [OR (95% CI): 0.93 (0.69–1.25), *p* = 0.631] of severe COVID-19 infection on eclampsia (Figure 3 and Supplementary Table S2).

### Causal effects of hospitalized COVID-19, on pre-eclampsia and eclampsia

Using IVW analysis as the primary approach, we found evidence of a suggestive causal effect of genetic liability to hospitalized COVID-19 infection on the risk of PE [OR (95% CI): 1.10 (1.01–1.19), *p* = 0.026], with odds ratios greater than 1 across the other MR methods. However, IVW analysis indicated no significant causal effect [OR (95% CI): 0.90 (0.61–1.31), *p* = 0.572] of severe COVID-19 infection on eclampsia (Figure 3 and Supplementary Table S2).

### Causal effects of general SARS-CoV-2 infection on pre-eclampsia and eclampsia

IVW analysis indicated no significant causal effects of general SARS-CoV-2 infection on either PE or eclampsia [OR (95% CI): 1.15 (0.88–1.52), *p* = 0.311, and OR (95% CI): 0.44 (0.18–1.11), *p* = 0.081, respectively] (Figure 3 and Supplementary Table S2).

The results of the rest MR analyses are presented in Supplementary Table S2.

### Scatter plots

The scatter plots displayed the effect of the IVs of three COVID-19 infection severity phenotypes on PE and eclampsia in the MR analysis. A positive correlation was indicated by a slope greater than zero and *vice versa* (Supplementary Figure S1).

### Sensitivity analysis

The robustness of the aforementioned causal relationships was confirmed by the results of sensitivity analysis (Supplementary Table S3). Heterogeneity testing yielded negative results in the MR analyses (Cochran's *Q*-statistic, *p*-values >0.05), which means the difference in the effect of SNPs between each pair of GWAS samples was sufficiently small to conduct a two-sample Mendelian randomization study. MR-Egger regression did not indicate significant horizontal pleiotropy (*p*-values >0.05 for the MR-Egger intercept). The MR-PRESSO global test indicated no significant outliers driving the causal effects (*p*-values >0.05). Due to the limited number of SNPs, it was difficult to assess sensitivity through funnel plots (Supplementary Figure S2). In addition, leave-one-out analyses indicated that no single SNP determined the causal effects alone (Supplementary Figure S3). The causal effect of each single SNP was demonstrated as forest plots (Supplementary Figure S4) and tables (Supplementary Table S4), with rs111837807 (attributed to critical ill COVID-19 and hospitalized COVID-19), rs41264915 (attributed to hospitalized COVID-19) exhibiting a suggestive causal effect on PE. The results of the Steiger test revealed that reverse causality was not validated across all selected IVs (see Supplementary Table S3).

The reverse causality referred to here means that the overall association between the instrumental variables and the outcome is stronger than with the exposure. This violates the exclusion restriction principle for valid instrumental variables, making it impossible to determine the causal direction.

## Discussion

Currently, most observational studies report that COVID-19 infection increases the risk of pre-eclampsia. However, due to differences in inclusion criteria, study populations, sample sizes, and covariates, the results vary across studies. A few observational studies suggest no significant association between COVID-19 infection and pre-eclampsia, while some studies account for COVID-19 severity and propose that only severe COVID-19 infection requiring oxygen support increases the risk of pre-eclampsia, whereas mild COVID-19 infection does not lead to pre-eclampsia. Most current observational studies do not stratify COVID-19 severity in pregnant women. The inconsistent conclusions from observational studies may result from the different distributions of COVID-19 severity across the study populations. To further investigate the relationship between COVID-19 infection and pre-eclampsia, we selected COVID-19 phenotypes with different severity levels and used MR to avoid biases from confounding factors and reverse causation inherent in traditional observational studies. In addition, given that PE can be screened early and prevented with low-dose aspirin, calcium supplementation, and moderate exercise (8), elucidating the relationship between COVID-19 and PE is critical. Through MR analyses, we found suggestive causal relationships between COVID-19 and PE but no significant causal association with eclampsia. Specifically, the COVID-19 phenotypes leading to critical illness and hospitalization were causally related to PE, while general SARS-CoV-2 infection did not have a significant causal relationship with PE. This indicates that COVID-19 may only increase the risk of PE when reaching a certain level of severity.

Our findings have clinically implicated that society needs to pay attention to SARS-CoV-2 infection among pregnant women and make efforts in COVID-19 prevention and early treatment to reduce the development of pre-eclampsia stemming from hospitalization and critical illness due to disease progression of SARS-CoV-2 infection. Especially for pregnant women hospitalized with COVID-19, measures need to be taken to prevent progression to critical illness to avoid an increased incidence of superimposed pre-eclampsia.

Our finding of a suggestive causal relationship between COVID-19 and PE is consistent with most previous observational studies and is pathophysiologically supported by findings from previous mechanistic studies. COVID-19 and PE share commonalities of vascular endothelial injury; thus, SARS-CoV-2 infection may mimic or exacerbate PE by causing potential microvascular endothelial damage and endotheliitis, leading to vasoconstriction and ischemia within the placenta. The mechanisms include the following:

- SARS-CoV-2 binding to the increased maternal ACE2 during pregnancy results in ACE2 downregulation, leading to an upregulated angiotensin II/angiotensin (1–7) ratio, resulting in vasoconstriction and hypertension (1);
- Expression of ACE2 by maternal endothelial cells makes them susceptible to SARS-CoV-2 infection, inducing complement activation and excessive C5b-9 complex formation, triggering coagulation pathway activation, and attracting neutrophils to form neutrophil extracellular traps (NETs), which express peptidylarginine deiminase; this reduces the activity of a disintegrin and metalloproteinase with thrombospondin type 1 repeats, member 13 (ADAMTS13), impairing its inhibition of platelet activation, provoking platelet activation and thrombotic microangiopathy (TMA), and eventually compromising placental perfusion and injuring vascular endothelium; decreased ADAMTS13 activity was also observed in severe PE (50); furthermore, elevated serum aPLA levels were reported in 52% of patients with COVID-19; among the aPLAs, anti-β2-glycoprotein-I, the primary pathogenic antibody in anti-phospholipid syndrome, also promotes complement activation, which facilitates thrombosis and inflammation in the placenta (51);
- SARS-CoV-2 can infect syncytiotrophoblast progenitors in the first trimester, activating placental inflammatory responses (52–54), and may interact with two placental proteins, TLE3 and LOX (55), leading to decidual arteriopathy including mural hypertrophy of the membrane, fibrinoid necrosis, and arterioles atherosis; this causes placental ischemia and endothelial cell dysfunction, which may give rise to PE (50, 56); the spike protein of SARS-CoV-2 was observed to upregulate transmembrane protease serine 2 (TMPRSS2) within syncytiotrophoblast and cytotrophoblast cells *in vitro*, increasing their susceptibility to SARS-CoV-2; it was also reported that the spike protein induced placental cells to produce pro-inflammatory cytokines and caused syncytiotrophoblast apoptosis *in vitro* (57);
- Due to the physiological downregulation of placental ACE2 expression in the second and third trimesters, SARS-CoV-2 is less likely to directly infect the placenta (54); however, it may indirectly damage it by provoking a distinct inflammatory response at the maternal–fetal interface (58).

Notably, the odds ratio was higher for the causal effect of critical ill COVID-19 on PE compared to hospitalized COVID-19. It was potentially due to exaggerated inflammatory responses and myocardial injury in severe COVID-19 cases. In severe COVID-19 infection, exaggerated inflammation manifests as a cytokine storm (59). The T-helper 1 (Th1)/T-helper 2 (Th2) immune shift in pregnant women during the first and second trimesters downregulates pro-inflammatory Th1-related cytokines and upregulates anti-inflammatory Th2-related cytokines (60), helping prevent cytokine storms (61). However, in the third trimester, the protective immune shift in pregnant women gradually disappears as Th2 polarization transitions toward a Th1/Th2 balance. With anti-inflammatory capabilities similar to non-pregnant women combined with all the respiratory and cardiovascular disadvantages of pregnancy, pregnant women in the third trimester are more prone to severe systemic inflammation, injuring maternal and placental vascular endothelium. Moreover, in severe COVID-19 cases, direct viral damage or inflammation-mediated injury to the myocardium can impair cardiac function. This myocardial impairment puts infected pregnant women at risk for cardiac disease and myocarditis. The resulting inadequate placental and organ perfusion, along with acquired ischemia, disturbs the equilibrium between factors promoting and inhibiting angiogenesis. This imbalance contributes to the pathogenesis of PE (62–65).

The observational study by Lai et al. also found a dose–response relationship between COVID-19 severity and PE risk, with severe and moderate COVID-19 infection increasing the risk of PE 5-fold and 3.3-fold, respectively, vs. asymptomatic cases, corroborating our findings (66). In addition, we did not find a significant relationship between general SARS-CoV-2 infection and PE, consistent with the findings of DuBose et al. that ordinary COVID-19 infection did not increase PE risk in pregnant women compared to non-infected women, while COVID-19 pneumonia did (24). The three distinct COVID-19 severity phenotypes demonstrated a stepwise increasing relationship with the risk of pre-eclampsia.

Compared to previous traditional observational studies stratifying COVID-19 severity, our study provides more rigorous evidence from the genetic susceptibility perspective. Therefore, it may help explain the inconsistent conclusions from previous observational studies. In addition to the contradiction between positive and negative findings mentioned previously, Papageorghiou's study (positive result) proposed that symptomatic vs. asymptomatic COVID-19 infection did not affect the risk increase for pre-eclampsia (67), conflicting with Lai et al.’s conclusion (COVID-19 severity was positively correlated with pre-eclampsia risk), who stratified COVID-19 severity into categories including (1) asymptomatic, (2) symptomatic without respiratory distress, (3) evidence of lower respiratory tract infection with SpO_2_ < 94%, and (4) requiring intensive care (66). Our results support Lai et al.’s conclusion from the genetic susceptibility perspective. Papageorghiou et al. did not report the proportion of pneumonia or severe cases in COVID-19-infected pregnant women. Since they did not further distinguish the severity of symptomatic COVID-19 infection, variation in the proportion of severe cases from different centers may introduce bias to their results.

Eclampsia develops from the exacerbation of pre-eclampsia. The non-significant association between COVID-19 and eclampsia could be due to the reduced incidence of eclampsia under modern medical care, where pre-eclampsia diagnosis and intervention prevent the exacerbation. Therefore, eclampsia patients captured in GWAS consist of both pre-eclampsia cases exacerbated after treatment and *de novo* eclampsia cases. The eclampsia-related SNPs thus represent the genetic susceptibility to both subtypes. If COVID-19 is not significantly associated with the “pre-eclampsia exacerbated into eclampsia” subtype, the overall non-significant result between COVID-19 and “eclampsia” could ensue in our study. In addition, as the incidence of eclampsia in the population is much lower than that of PE, GWAS studies generally include smaller sample sizes. The GWAS studies used in this research only included 290 eclampsia cases in the phenotype case group, and associated genetic mutations may differ from the general population. This could explain why none of the three COVID-19 severity phenotypes showed significant causal relationships with eclampsia in our study.

Since Mendelian randomization uses SNPs (i.e., mutation sites) merely as a tool for statistical analysis, the SNPs (IVs) themselves do not necessarily play an explicit bridging role between the two phenotypes. The variants actually taking effect could be other SNPs in linkage disequilibrium with the IVs. Therefore, in our discussion, we can only speculate about potential mechanisms behind the IVs that are causally related to the outcome. Concrete mechanisms need to be verified by experimental studies.

Previous studies support our findings regarding the causal effects of individual SNPs rs41264915 and rs111837807 on PE and eclampsia. SNP rs41264915 was reported to upregulate the level of TGF-β1, which is involved in both COVID-19 and PE pathophysiology. The upregulation of TGF-β1 mediates enhanced IgA responses, exacerbating the host inflammatory response brought on by SARS-CoV-2 and worsening the disease (68). Circulating TGF-β1 levels were also reported to be higher in women with both early- and late-onset PE compared to healthy pregnant women at diagnosis (69), and the potential mechanisms include the following:

- TGF-β1 may competitively bind transthyretin, reducing its absorption of soluble endoglin (sEng) in circulation that induces PE (70);
- TGF-β1 activates SMAD and ERK1/2 signaling pathways, leading to upregulation (71, 72);

The aforementioned pathways result in insufficient trophoblast cell invasion, thereby contributing to PE development.

SNP rs41264915 is located in THBS3 (39), whose mutation has been reported to increase its expression in various human tissues (including the lungs), leading to elevated levels of thrombospondins, which also upregulate the level of TGF-β1. By directly attaching to the dormant TGF-1 precursor complex, thrombospondins activate TGF-1, resulting in an increase in TGF-β1.

SNP rs111837807 is localized in gene CCHCR1 (39), which is found to encode a protein with five coiled-coil alpha-helical rod domains that regulate mRNA metabolism by interacting with mRNA-decapping protein 4 (73). However, research on the association between CCHCR1 and COVID-19 or PE is limited.

Increased PE risk from coronavirus infection has been observed before, with case series reports of MERS coronavirus inducing PE (74). The relationships between coronaviruses and PE should be considered in potential future epidemics.

Our study has some limitations. The generalizability of our findings to non-European ancestries is constrained, to a certain degree, by our primary inclusion of individuals of European descent to mitigate racial influences. Applicable genetic evidence from Asian, American, and African populations is needed to enact research on such causal associations and elucidate potential genetic variances between ethnicities. In addition, the utilization of aggregated summary data rather than individual-level statistics for our MR impedes subgroup examinations based on gestational period, COVID-19 infection history, infected SARS-CoV-2 variant, vaccination records, etc.

Pre-eclampsia-like syndrome (PE-like syndrome) has been reported to be induced by severe pneumonia caused by COVID-19, and it may recover concurrently with the remission of pneumonia without the need for planned delivery. Maintenance of normal values for the serum-soluble FMS-like tyrosine kinase 1 to placental growth factor ratio and the uterine artery pulsatility index (UtAPI) is characteristic of patients with this pathology, even in the presence of hypertension and other PE-like manifestations (75, 76). An observational study (sample size = 68, number of cases = 15) suggested that approximately half of pre-eclampsia-like syndrome cases may be misdiagnosed as pre-eclampsia (76). However, constrained by the laboratory and ultrasonographic examinations required to differentiate these two pathologies, most observational studies, including the FinnGenn project, did not distinguish between PE-like syndrome and PE syndrome (22, 77, 78). Hence, the results regarding the association between critical COVID-19 illness and pre-eclampsia in this study may be impacted. However, while relevant observational studies reported no cases of PE-like syndrome in non-severe pneumonia hospitalized cohorts, we still detected a causal relationship between hospitalized COVID-19 and pre-eclampsia (75, 76). This denotes that such association persists irrespective of PE-like syndrome.

Regarding future research directions in this field, we suggest the following: validating this causal relationship in different ethnicities; investigating the impacts of COVID-19 infection history, vaccination history, obstetric history and timing of vaccination, COVID-19 infection variants, etc., on this causal relationship based on individual-level data; assessing whether aspirin prevents pre-eclampsia following COVID-19 infection and whether the preventive effect relates to COVID-19 severity and its safety; determining the optimal timing for initiating aspirin; and investigating the mechanisms underlying COVID-19-induced hypertensive disorders in pregnancy and potential interventional targets.

According to our findings, we recommend that pregnant women and their families wear masks when going out to prevent COVID-19 infection. For pregnant women with mild COVID-19 infection, no special screening for pre-eclampsia is needed. For hospitalized and severe COVID-19-infected pregnant women, enhanced monitoring for pre-eclampsia is recommended, including weekly monitoring of blood pressure and proteinuria. Active intervention should be given immediately if there are signs of pre-eclampsia. Pregnant women infected with COVID-19 can take oral calcium supplements to prevent pre-eclampsia, especially in areas with low dietary calcium intake. The dose of calcium supplements should be at least 1 g/day (79).

## Conclusions

We found that genetic susceptibility to critical ill COVID-19 and hospitalized COVID-19 may lead to an increased risk of pre-eclampsia, while the causal relationship with eclampsia risk was not significant. Genetic predisposition to general SARS-CoV-2 infection did not exhibit significant causal relationships with risks of pre-eclampsia or eclampsia. The COVID-19 severity demonstrated a potential positive suggestive dose–response relationship with the risk of pre-eclampsia. More attention should be paid to pregnant women hospitalized for COVID-19 for prevention and screening of PE, especially those needing respiratory support.

## Data Availability

The original contributions presented in the study are included in the article/Supplementary Material, further inquiries can be directed to the corresponding authors.
