# Supplementary material for: Mendelian randomization supports genetic liability to hospitalization for COVID-19 as a risk factor of pre-eclampsia
Source: Front Cardiovasc Med. 2024 Mar 8;11:1327497. doi: 10.3389/fcvm.2024.1327497 (PMC10957568; doi:10.3389/fcvm.2024.1327497)
Supplement: Supplementary file 1 [file Image1.pdf]

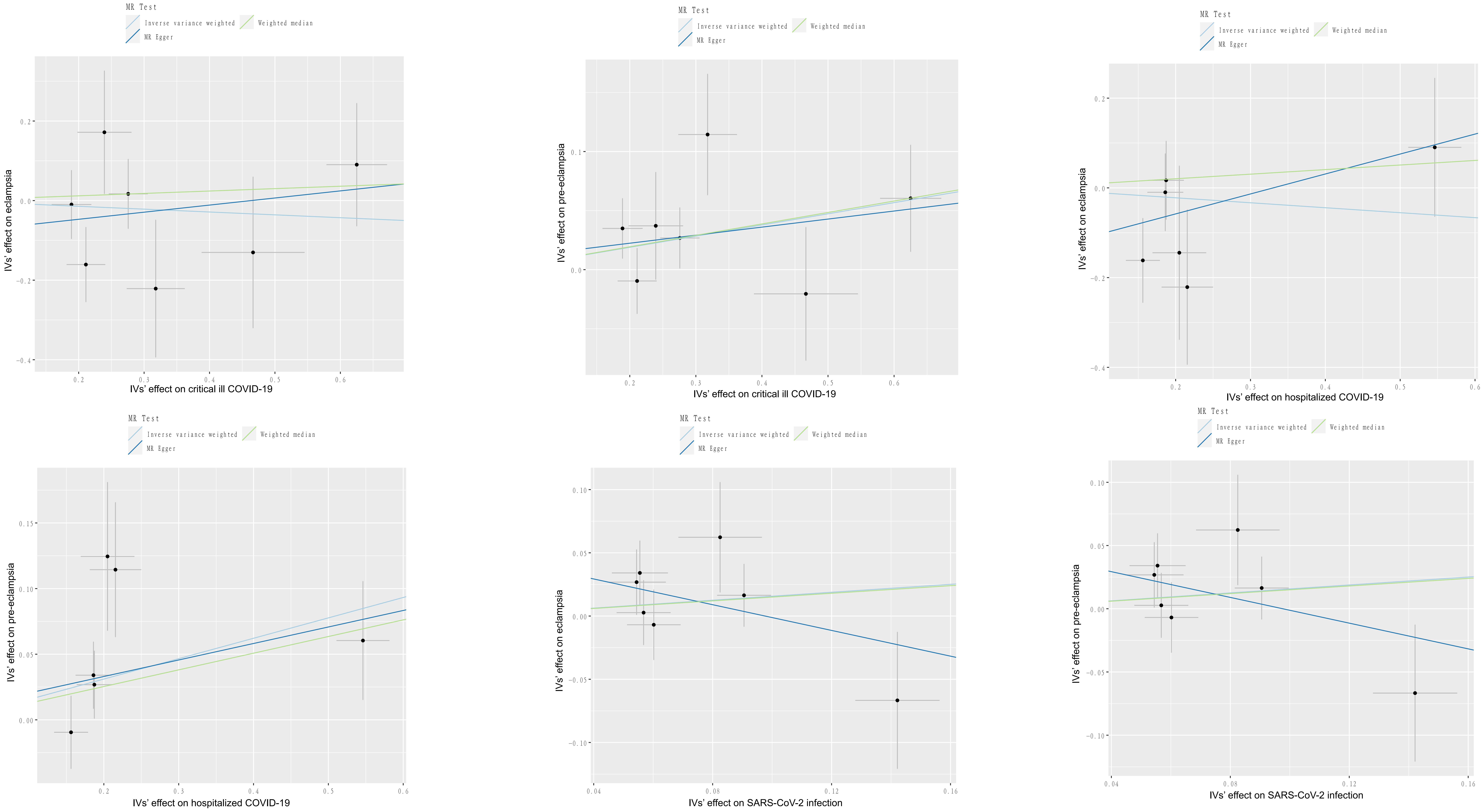

Supplement Figure 1 Scatter plots of the effect of instrumental variables of three COVID-19 severity phenotypes on pre-eclampsia and eclampsia. Abbreviations: IVs - instrumental variables.
