# Supplementary material for: Mendelian randomization supports genetic liability to hospitalization for COVID-19 as a risk factor of pre-eclampsia
Source: Front Cardiovasc Med. 2024 Mar 8;11:1327497. doi: 10.3389/fcvm.2024.1327497 (PMC10957568; doi:10.3389/fcvm.2024.1327497)
Supplement: Supplementary file 2 [file Image2.pdf]

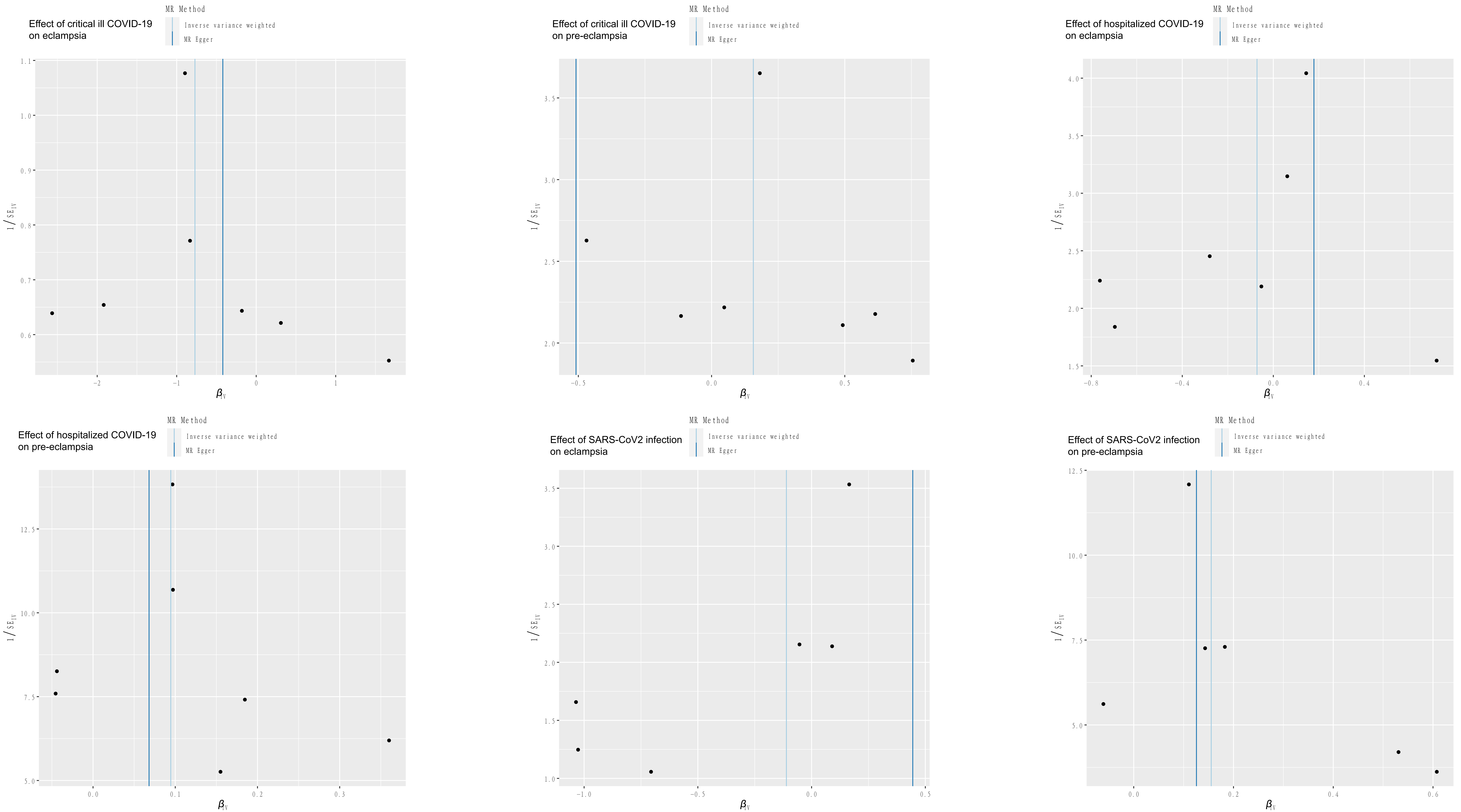

Supplement Figure 2 Funnel plots of effects of three types of COVID-19 traits on pre-eclampsia and eclampsia. Abbreviations: MR - mendelian randomization; SE - standard error.
