# Supplementary material for: Mendelian randomization supports genetic liability to hospitalization for COVID-19 as a risk factor of pre-eclampsia
Source: Front Cardiovasc Med. 2024 Mar 8;11:1327497. doi: 10.3389/fcvm.2024.1327497 (PMC10957568; doi:10.3389/fcvm.2024.1327497)
Supplement: Supplementary file 3 [file Image3.pdf]

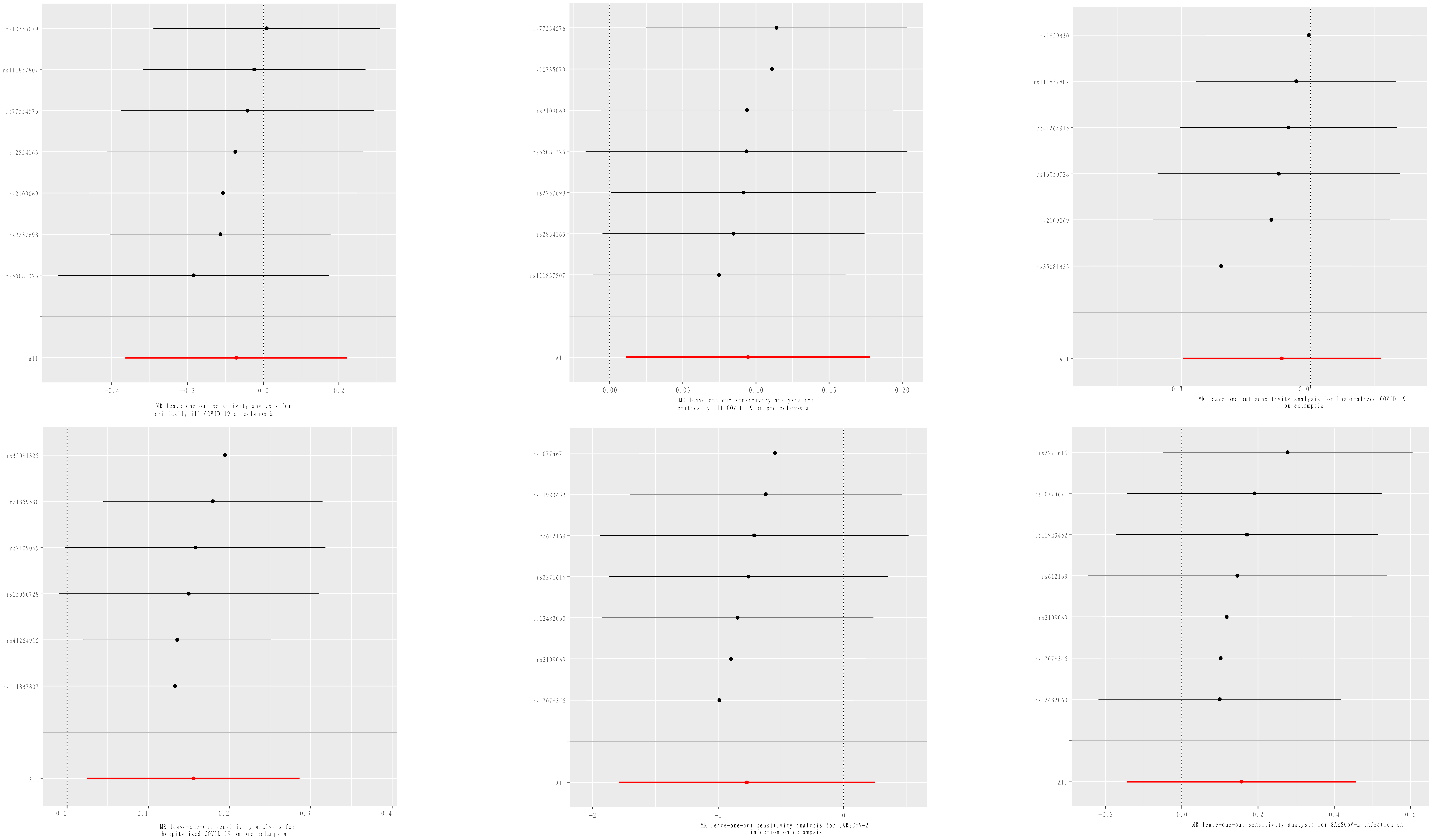

Supplement Figure 3 Leave-one-out analyses of causal effect of three COVID-19 severity phenotypes on pre-eclampsia or eclampsia.
