# Supplementary figures and images for: Mendelian randomization supports genetic liability to hospitalization for COVID-19 as a risk factor of pre-eclampsia
Source: Front Cardiovasc Med. 2024 Mar 8;11:1327497. doi: 10.3389/fcvm.2024.1327497 (PMC10957568; doi:10.3389/fcvm.2024.1327497)

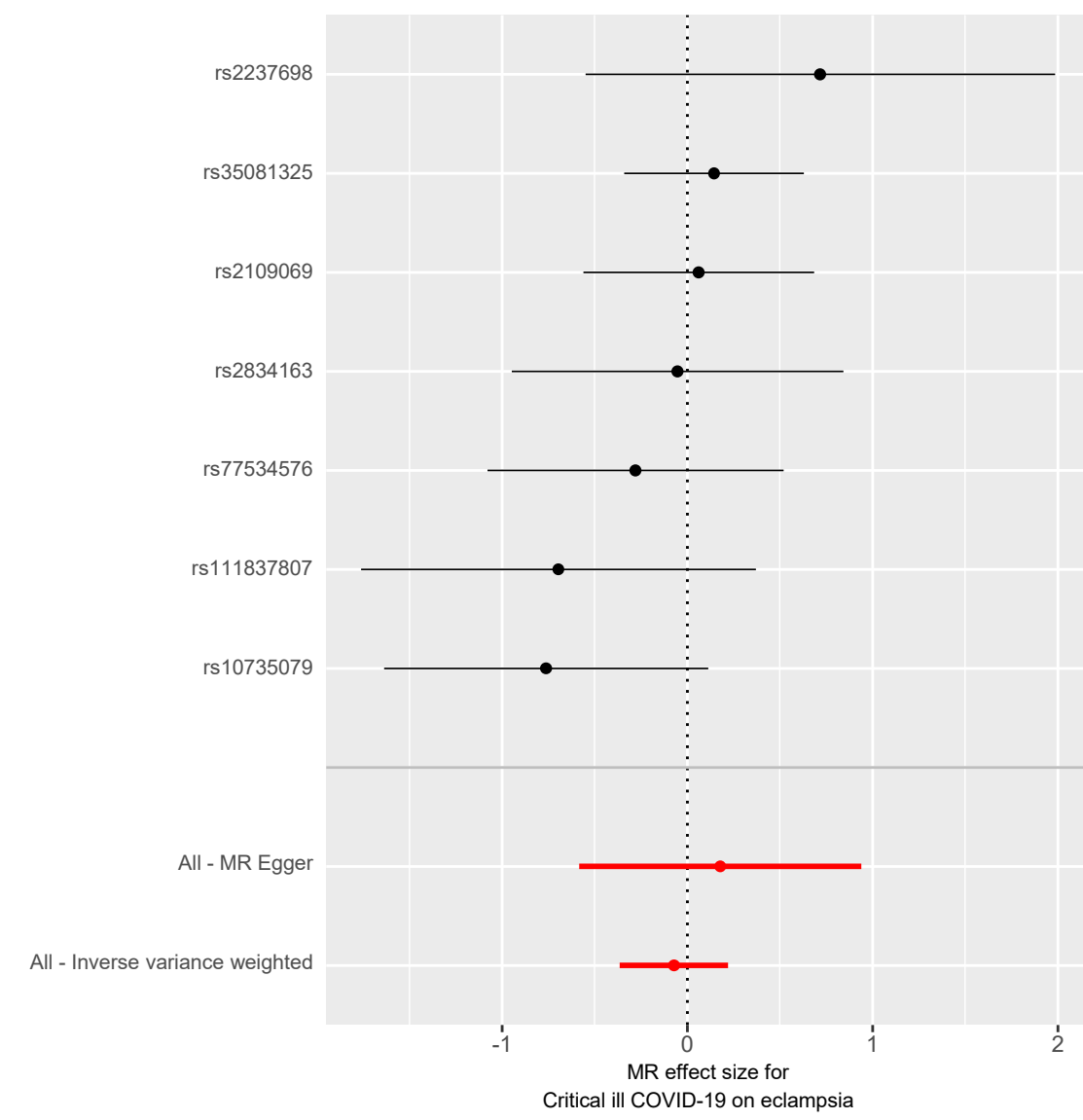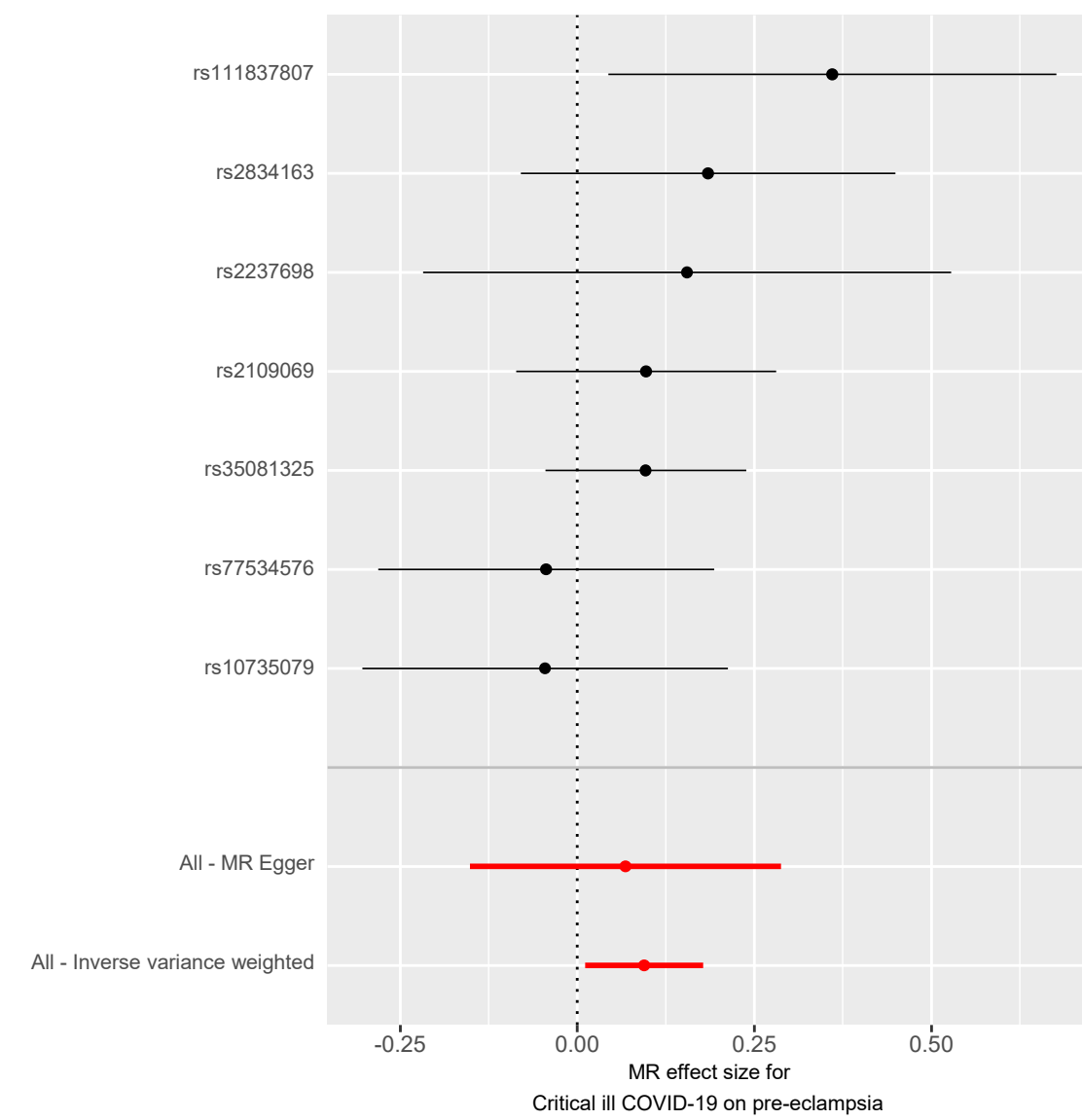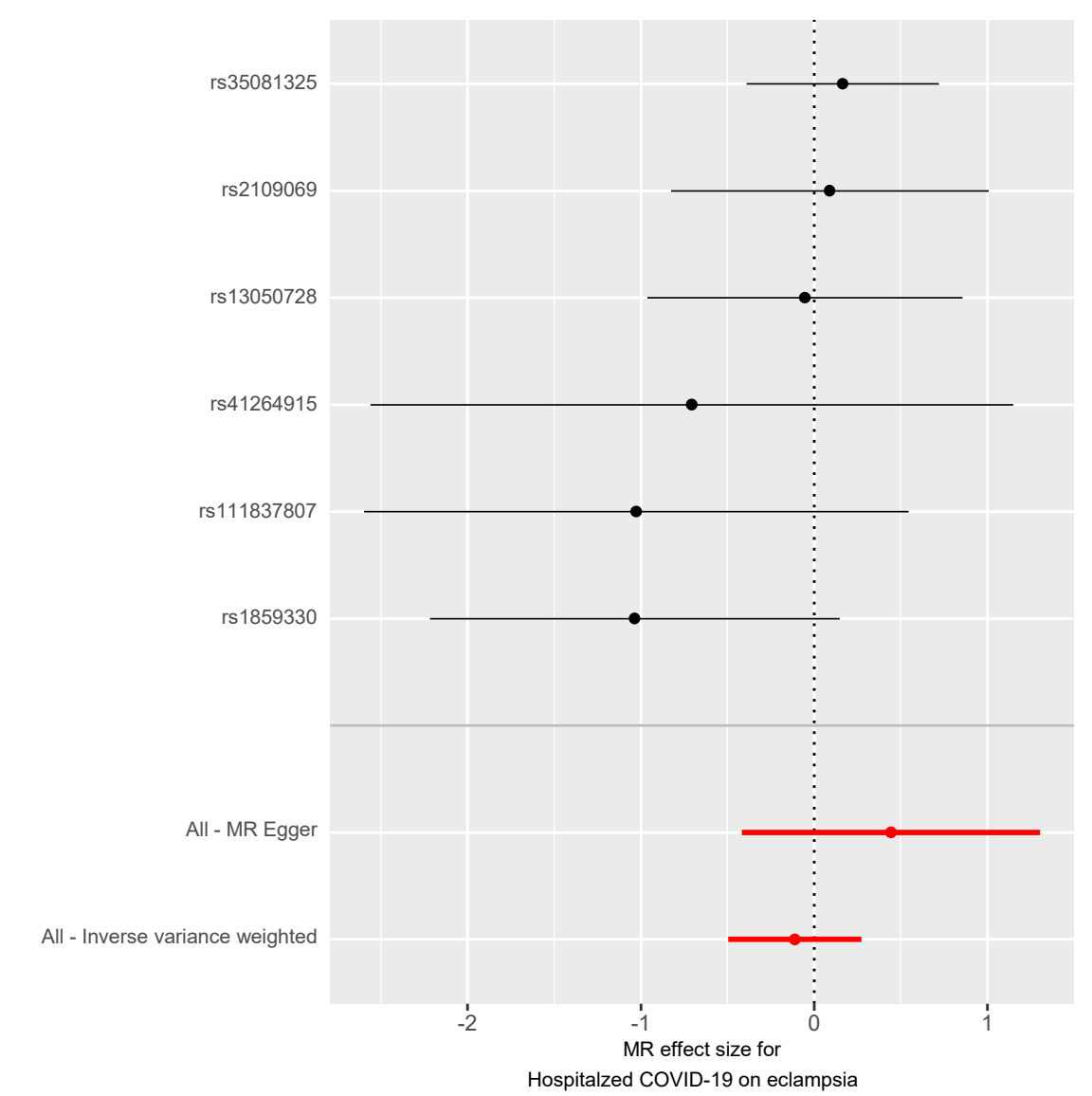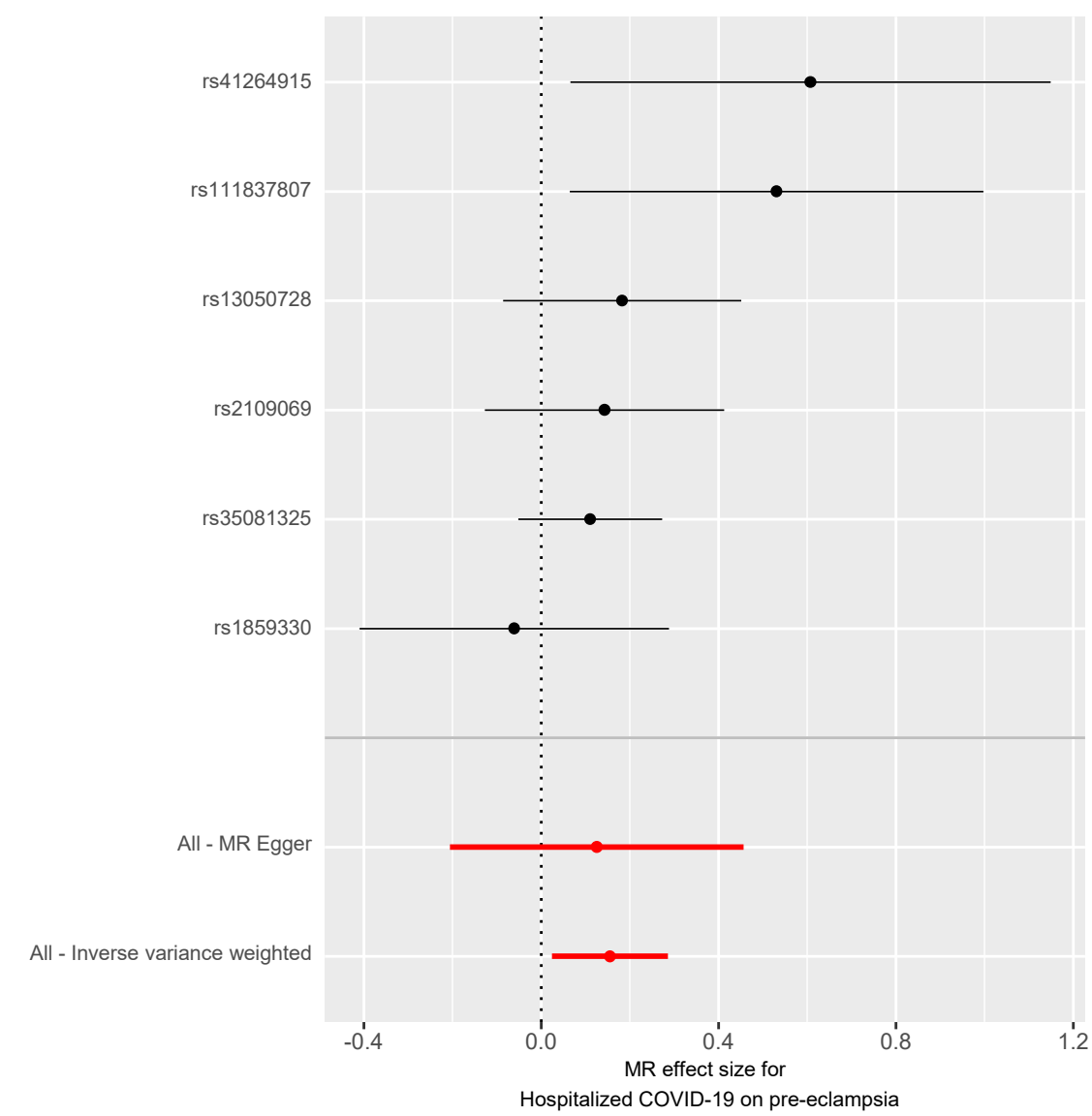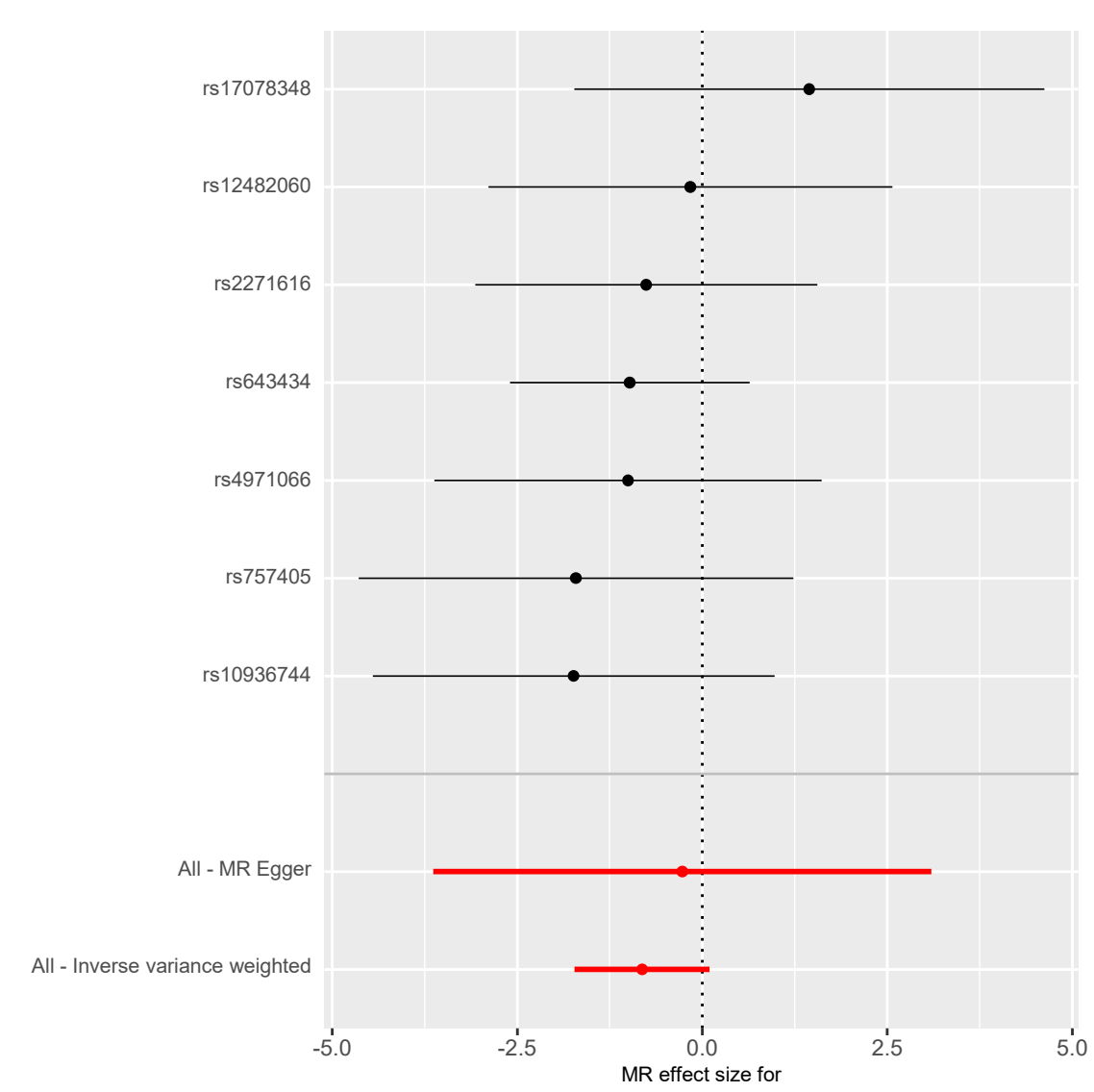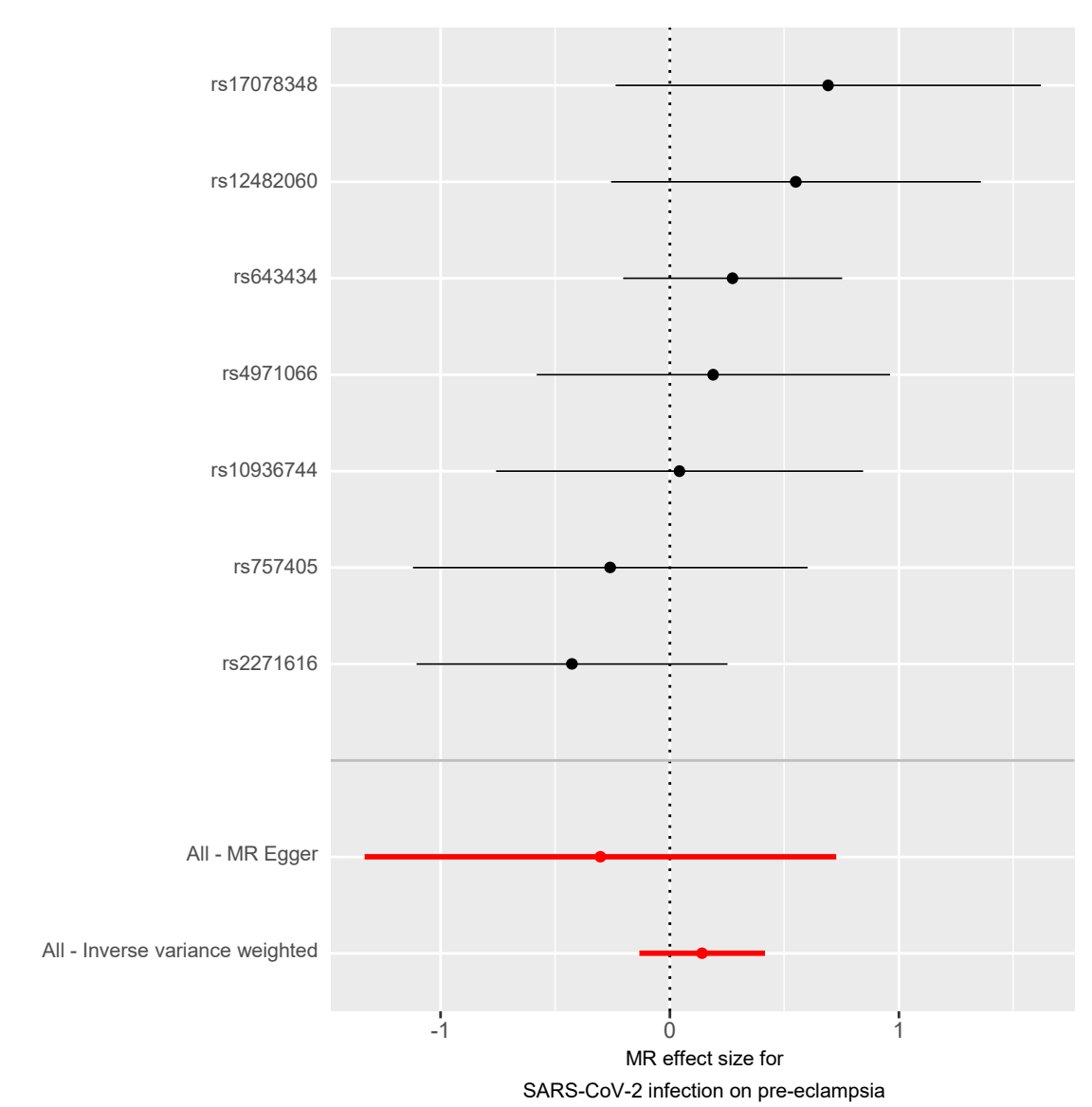

Supplement Figure 4 Causal effect of each single SNP on pre-eclampsia and eclampsia.

Supplement: Supplementary file 4 [file Image4.pdf]
